# Supplementary material for: Genetic and pharmacological evidence for kinetic competition between alternative poly(A) sites in yeast
Source: eLife. 2021 Jul 7;10:e65331. doi: 10.7554/eLife.65331 (PMC8263057; doi:10.7554/eLife.65331)
Supplement: Transparent reporting form [file elife-65331-transrepform.docx]

***eLife’s* transparent reporting form**

We encourage authors to provide detailed information *within their submission* to facilitate the interpretation and replication of experiments. Authors can upload supporting documentation to indicate the use of appropriate reporting guidelines for health-related research (see [EQUATOR Network](http://www.equator-network.org/%20)), life science research (see the [BioSharing Information Resource](https://biosharing.org/)), or the [ARRIVE guidelines](http://www.plosbiology.org/article/info:doi/10.1371/journal.pbio.1000412) for reporting work involving animal research. Where applicable, authors should refer to any relevant reporting standards documents in this form.

If you have any questions, please consult our Journal Policies and/or contact us: [editorial@elifesciences.org](mailto:editorial@elifesciences.org).

**Sample-size estimation**

- You should state whether an appropriate sample size was computed when the study was being designed
- You should state the statistical method of sample size computation and any required assumptions
- If no explicit power analysis was used, you should describe how you decided what sample (replicate) size (number) to use

Please outline where this information can be found within the submission (e.g., sections or figure legends), or explain why this information doesn’t apply to your submission:

No statistical tools were used for sample size estimation. For NGS, two replicates are generally used in the yeast studies because it is a highly robust system. Here, two replicates were chosen as a suitable number because of the degree of internal replication between strains and/or over a time-course of sampling. The robustness of the system was independently validated prior to high-content data collection.

For Proteomics and Metabolomics 5 replicates were chosen based on the expertise of RBS and DJC respectively.

**Replicates**

- You should report how often each experiment was performed
- You should include a definition of biological versus technical replication
- The data obtained should be provided and sufficient information should be provided to indicate the number of independent biological and/or technical replicates
- If you encountered any outliers, you should describe how these were handled
- Criteria for exclusion/inclusion of data should be clearly stated
- High-throughput sequence data should be uploaded before submission, with a private link for reviewers provided (these are available from both GEO and ArrayExpress)

Please outline where this information can be found within the submission (e.g., sections or figure legends), or explain why this information doesn’t apply to your submission:

All replication data is provided in the methods/main text

NGS samples were performed in biological replicate where each yeast strain grown/treated for experimental sampling from separate colonies grown on different days.

For Proteomics and Metabolomics 5 replicates (as above) were sampled from separate cultures grown on separate days. In the metabolomics data, one sample from each timepoint was excluded during analysis due to inadvertent phase separation (possibly due to residual water carryover)

For pharmacological treatments (Figure 6C) the data shown are representative of two independent repeats (sampled and analyzed on separate days)

For Seahorse analysis (Sup Figure 3) data shown are representative of 3 independent experiments.

**Statistical reporting**

- Statistical analysis methods should be described and justified
- Raw data should be presented in figures whenever informative to do so (typically when N per group is less than 10)
- For each experiment, you should identify the statistical tests used, exact values of N, definitions of center, methods of multiple test correction, and dispersion and precision measures (e.g., mean, median, SD, SEM, confidence intervals; and, for the major substantive results, a measure of effect size (e.g., Pearson's r, Cohen's d)
- Report exact p-values wherever possible alongside the summary statistics and 95% confidence intervals. These should be reported for all key questions and not only when the p-value is less than 0.05.

Please outline where this information can be found within the submission (e.g., sections or figure legends), or explain why this information doesn’t apply to your submission:

Information concerning the statistical tests used is found in the sections, figure legends and described in full in the methods.

(For large datasets, or papers with a very large number of statistical tests, you may upload a single table file with tests, Ns, etc., with reference to sections in the manuscript.)

**Group allocation**

- Indicate how samples were allocated into experimental groups (in the case of clinical studies, please specify allocation to treatment method); if randomization was used, please also state if restricted randomization was applied
- Indicate if masking was used during group allocation, data collection and/or data analysis

Please outline where this information can be found within the submission (e.g., sections or figure legends), or explain why this information doesn’t apply to your submission:

N/A

**Additional data files (“source data”)**

- We encourage you to upload relevant additional data files, such as numerical data that are represented as a graph in a figure, or as a summary table
- Where provided, these should be in the most useful format, and they can be uploaded as “Source data” files linked to a main figure or table
- Include model definition files including the full list of parameters used
- Include code used for data analysis (e.g., R, MatLab)
- Avoid stating that data files are “available upon request”

Please indicate the figures or tables for which source data files have been provided:

All NGS data is available in GEO with the accession number GSE160539 (reviewer token eponueowrzofluv).

Significant APA genes in figure 1D, 2A and 4B are listed in supplemental file 2

Proteomics data are available in supplemental file 3

Metabolomics data are available in supplemental file 4

Genes lists used for Figure 7 are available in supplemental file 5
